# Genome-Wide Identification and Evolutionary Analysis of the Animal Specific ETS Transcription Factor Family

**DOI:** 10.4137/ebo.s2948

**Published:** 2009-10-02

**Authors:** Zhipeng Wang, Qin Zhang

**Affiliations:** State Key Laboratory for Agrobiotechnology, Key Laboratory of Animal Genetics and Breeding of the Ministry of Agriculture, College of Animal Science and Technology, China Agricultural University, Beijing, 100193, China. Email: qzhang@cau.edu.cn

**Keywords:** ETS transcription factors, metazoan animal, genome-wide, phylogenetic analysis

## Abstract

The ETS proteins are a family of transcription factors (TFs) that regulate a variety of biological processes. We made genome-wide analyses to explore the classification of the ETS gene family. We identified 207 ETS genes which encode 321 ETS TFs from ten animal species. Of the 321 ETS TFs, 155 contain only an ETS domain, about 50% contain a ETS_PEA3_N or a SAM_PNT domain in addition to an ETS domain, the rest (only four) contain a second ETS domain or a second ETS_PEA3_N domain or an another domain (AT_hook or DNA_pol_B). A Neighbor-Joining phylogenetic tree was constructed using the amino acid sequences of the ETS domain of the ETS TFs. The results revealed that the ETS genes of the ten species can be divided into two distinct groups. Group I contains one nematode ETS gene and 18 vertebrate animal ETS genes. Group II contains the majority of the ETS TFs and can be further divided into eleven subgroups. The sequence motifs outside the DNA-binding domain and the conservation of the exon-intron structural patterns of the ETS TFs in human, cattle, and chicken further support the phylogenetic classification among these ETS TFs. Extensive duplication of the ETS genes was found in the genome of each species. The duplicated ETS genes account for ~69% of the total of ETS genes. Furthermore, we also found there are ETS gene clusters in all of the ten animal species. Statistical analysis of the Gene Ontology annotations of the ETS genes showed that the ETS proteins tend to be related to RNA biosynthetic process, biopolymer metabolic process and macromolecule metabolic process expected from the common GO categories of transcriptional factors. We also discussed the functional conservation and diversification of ETS TFs.

## Introduction

Transcription factors (TFs) are the key regulators of gene expression at the transcriptional levels. They play crucial roles in the life cycle or biological processes of all living organisms, such as development, growth, and responses to environmental stimulus. TFs are usually classified into different families and subfamilies based on the sequence of DNA-binding domains they contain, which are highly conserved among species.^1^,^2^ Some of these families are common to most eukaryotic organisms, and some are specific to a given taxonomic group.

The ETS TF family is one of the largest families of TFs. All members of this family share a highly conserved DNA-binding domain of 85 amino acid residues named the ETS domain. The ETS family is further sub-classified into a number of subfamilies 3,4 based on the sequence similarities of the ETS domain and the presence of additional conserved domains. The ETS TFs are present throughout the body and are involved in a wide variety of functions including the regulation of cellular differentiation, cell cycle control, cell migration, cell proliferation, apoptosis (programmed cell death), and angiogenesis.^4^ Many ETS TFs have been found to be associated with cancer through gene fusion. For example, the fusion of TEL to the JAK2 protein results in early pre-B acute lymphoid leukaemia.^4^ Some ETS TFs appear to regulate positively or negatively other transcription factor activities.^5^ This allows combinatorial control of gene expression and enhances the action specificity the ETS-domain proteins. For example, Ets-1 interacts with bHLH proteins,^6^ which activates transcription regulation and enhances DNA binding. Since the ETS TFs are important factors in the network of protein-protein interactions that govern transcription regulation, identifying the extent of ETS TFs and the subfamilies they belong to at the genome-wide level is an important step to understand the gene regulatory network.

With genome sequence data for more and more species becoming available, it is now possible to compare the ETS genes among different animal species at the genome-wide level. Determining the phylogenetic relationships of the ETS genes is an important step for elucidating the evolutionary and functional divergence of this gene family. Phylogenetic analyses have been conducted for many other TF families including the bHLH family,^7^–^11^ the homeobox family,^12^ the nuclear receptor family,^13^ the WRKY family,^14^,^15^ the MADS family,^16^,^17^ the GATA family,^18^ the AP2 family,^19^ the DOF family,^20^,^21^ the SBP-box family,^22^,^23^ the heat shock family,^24^ the ERF family,^25^ the NF-Y family,^26^ the basic leucine zipper family,^27^ the Sox family,^28^ and the CCCH zinc finger family.^29^ As for the ETS family, Laudet^30^ studied the evolutionary relationship between the ETS genes using sequences of all known ETS family members extracted from the EMBL data library, Genbank, NBRF, and the Infobiogen network (www.infobiogen.fr). They showed that the genes of the ETS family can be divided into 13 groups namely ETS, ER71, GABP, PEA3, ERG, ERF, ELK, DETS4, ELF, ESE, TEL, YAN, and SPI.

In this study, we first collected all putative ETS TFs from 10 species and classified them into subfamilies based on their ETS domains. Then, we performed phylogenetic analysis to explore the evolutionary relationship of these ETS genes. The features of the gene structures, the patterns of the conserved motifs, and the function divergence were discussed as well.

## Materials and Methods

### Identification of ETS TFs and ETS genes

From the DBD database,^31^ we identified all putative TFs at the genome-wide level of ten species of the animal kingdom, one or two from each genus, for which the full genome sequences are available. The ten species are human (*Homo Sapiens*), mouse (*Mus musculus*), rat (*Rattus norvegicus*), cattle (*Bos Taurus*), chicken (*Gallus gallus*), sea squirt (*Ciona intestinalis*), frog (*Xenopus tropicalis*), zebrafish (*Danio rerio*), fruit fly (*Drosophila melanogaster*), and nematode (*Caenorhabditis elegans*). Then, we collected the proteome sequences of all these TFs from the Flybase database (for fruit fly) (http://flybase.org/, 32 and the ENSEMBL database release 47 (for all other species) (ftp://ftp.ensembl.org/pub/release-47/fasta/,).^33^,^34^ We performed the HMMER (http://hmmer.wustl.edu,)^35^ search for the ETS domain in the sequences using the profile PF00178 of the Pfam database (http://pfam.sanger.ac.uk/,)^36^ and results manually to obtain the ETS TFs. The genes encoding these ETS TFs were identified according to their annotation information. In addition, we collected the genome sequences of all identified ETS genes from the two databases.

### Phylogenetic analysis

For phylogenetic analysis, we considered only the amino acid sequences of the DNA-binding domains, i.e. the ETS domain, in the ETS TFs which exist in all or most species. For a gene with more than one splicing-isoforms, we retained only the longest sequence encoded by it. We used ClustalX (v1.81)^37^ for multiple sequence alignment with default settings and manually refined the alignment by removing the common gaps of some sequences. We used PhyML (v3.0)^38^ to construct the maximum likelihood (ML) phylogenetic trees with 1000 replicate bootstrap tests and set a cut-off bootstrap value of 65 to define clades in the ML trees. Representations of the calculated trees were constructed using MEGA (v4.0).^39^

### Exon-intron structure and motif analysis

The diagrams of the exon-intron structures of the ETS genes were obtained from the ENSEMBL database (http://www.ensembl.org/). The sequence logos were generated using the online platform Weblogo (http://weblogo.berkeley.edu/).^40^ We got the conserved motifs of the ETS proteins using the online platform MEME (http://meme.sdsc.edu/meme).^41^

### GO enrichment analysis

The gene ontology (GO) hierarchy annotations were downloaded from the Gene Ontology database (http://www.geneontology.org/). The enrichment of the GO categories was analyzed using the tool DAVID (http://david.abcc.ncifcrf.gov/home.jsp).^42^ DAVID calculates the functional enrichment score of the same gene set based on the GO categories including biological process, molecular function and cellular component. In addition to a p value, it also provides a FDR (false positive rate) value for each enrichment score. A FDR value of 0.05 was used as the significance threshold for defining a GO term.

## Results

### ETS subfamilies and distributions of ETS TFs in different subfamilies and in the ten species

We identified a total of 321 ETS TFs from the ten species, which are encoded by 207 genes. Of the 321 ETS TFs 155 contain only an ETS domain, about 50% contain a ETS_PEA3_N or a SAM_PNT domain in addition to an ETS domain, the rest (only four) contain a second ETS domain or a second ETS_ PEA3_N domain or an another domain (AT_hook or DNA_pol_B). We classified the ETS TFs into seven subfamilies according to the domain combinations they contain. The distributions of the ETS TFs in different subfamilies and in the ten species are shown in Table 1. Subfamilies ETS and ETS&SAM_PNT exist in all of the ten species. Subfamily ETS&ETS_ PEA3_N exists in 7 species. The other subfamilies exist only in one species. The number of ETS TF proteins (genes) varies in different species, from 11 (10) in nematode to 71 (29) in human. However, the proportions of the number of ETS TF proteins (genes) to the total number of TF proteins (genes) are very similar in all species, ranging from 2% ~3%, except in sea squirt (about 4%) and in nematode (about 1%).

### Phylogenetic relationship of ETS genes in the ten species

Three ETS subfamilies, ETS, ETS&ETS_PEA3_N, and ETS&SAM_PNT, which distribute in all or most of the ten species and correspond to 203 ETS genes (Table 1), were used to construct the phylogenetic tree. We designate these genes as “aabbbn”, where “aa” is the abbreviation of the species (hs for human, bt for cattle, mm for mouse, etc); “bbb” refers to the subfamily (ets for subfamily ETS, pea for subfamily ETS&ETS_PEA3_N, sam for subfamily ETS&SAM_PNT), “n” refers to the sequence number of the ETS gene in this subfamily. For example, hsets1 refers to the first gene of subfamily ETS in human. The detailed information of these genes is given in Supplemental Table S1.

**Figure 1:**
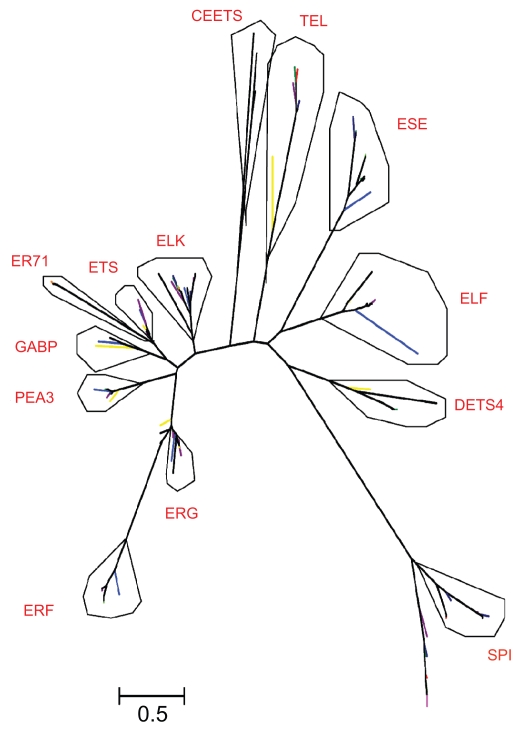
Unrooted ML trees of the ETS genes based on the amino acid sequences of the ETS-domain of all ETS TFs. The scale bar corresponds to 0.5 amino acid substitutions per residue. Different colors denote different species, red: human, magenta: mouse, orange: rat lime: cattle, green: chicken, blue: frog, darkblue: sea squirt, purple: zebrafish, yellow: fruit fly, black: nematode.

To resolve the phylogentic relationship between the ETS family members, we constructed an unrooted Maximum llikelihood (ML) phylogenetic tree (Fig. 1, Supplemental Fig. S3) for the 203 EST TFs from the 10 species based on the amino acid sequences of their ETS-domain. Of the 203 EST TFs, 197 were classified into two groups in the ML tree. The other 7, drets7, xtets11. ggets4, hsets14, btets8, rnets2, and mmets2, which were not able to be classified into these two groups, were independent from each other and hence removed from the ML tree. In the ML tree, Group I contains one nematode ETS TF and 11 vertebrate animal ETS TFs which can be divided into two clades. Group II contains 184 ETS TFs which are further divided into 12 subgroups. This classification is in general consistent with that of Laudet et al^30^ with minor difference (see Discussion). Following their nomenclature, we named group I SPI, and the 11 subgroups of group II, which are in common with their classification, ESE, TEL, ELF, DETS4, PEA3, ELK, ETS, ER71, GABP, ERF, and ERG. The other subgroup containing three nematode genes, ceets1, ceets4, and ceets8, which were not included in Laudet et al,^30^ was named CEETS. All members in group SPI contain only an ETS domain. Members in subgroups ELF, ELK, ER71, and ERF contain only the ETS-domain except cisam8 in ELF which contain the ETS and SAM-PNT domains. About 1/5 members in ERG contain only the ETS-domain and the rest contain the ETS and SAM_PNT domains. Members in PEA3 contain the ETS and ETS_PEA3_N domains except ciets2 and dmets4 which contain only the ETS domain. Members in ESE, TEL, DETS4, ETS, and GABP contain the ETS and SAM_PNT domain except dmets5, drets9, and xtets10 in ETS which contain only the ETS domain. In addition, using the ME (minimum evolution) and NJ (Neighbor-Joining) methods, we obtained trees with similar topology (data not shown). But in the ME tree, the subgroups PEA3, ELK, ETS, ER71, GABP, ERF, and ERG in the NJ and ML trees were merged into one group.

Based on the topology structure of the phylogenetic tree, we classified the ETS genes into four categories in the same way of Xiong et al,^43^ i.e. one-to-one category in which a gene in one species and its corresponding gene in the other species have a common ancestor, many-to-many category in which gene duplication occurred in one or some lineages, lineage-specific expansion category which includes clades that have two or more genes in one lineage and no gene in other lineages, and other. We then constructed the phylogenetic trees for three pairs of species, i.e. cattle and chicken, mouse and rat, and human and nematode, respectively. Based on these phylogenetic trees, we estimated the number of ancestral genes for each pair of species. For example, for the phylogenetic tree of cattle and chicken, there are 17 ancestral genes. From the phylogenetic tree for mouse and rat, we found all ETS genes in the two species are derived from 27 ancestral genes except genes rnpea1, mmpea1, and mmpea3.

### Sequence logos

**Figure 2:**
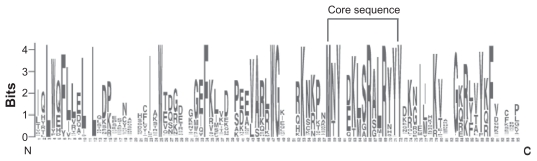
The whole sequence logo of the ETS domain and its core conserved sequence MAY(DE) KLSR(GA)LRYYY. The over all height of each stack indicated the sequence conservation at that position, whereas the height of symbols within each stack reflects the relative frequency of the corresponding amino acid.

The ETS domain in the ETS TFs is necessary for the specific recognition of a purine-rich core sequence GGAA/T flanked by more variable but not random 5′ and 3′ sequences.^44^,^45^ The most conserved part of the ETS domain is the sequence MNY(DE)KLSR(GA)LRYYY (Fig. 2). However, considerable variation in this sequence was observed among different ETS subgroups in the ML tree (Table 2). Such variation may have relation to the subgroup-specific functions of the ETS proteins. Indeed, the alteration of a single amino acid at the carboxy-terminal end of the DNA-recognition helix in the ETS domain can markedly alter its DNA-binding transcription factors.^46^,^47^

### Genomic distribution and duplication of ETS genes

**Figure 3:**
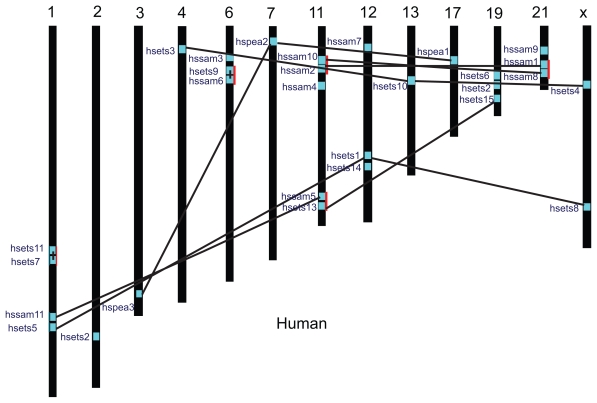
Distribution of ETS genes on the chromosomes of human. Chromosome numbers are indicated at the top of each bar. The small blue box on the chromosome indicated the position of the ETS gene with its name beside it. The duplicated ETS genes (either on different chromosomes or residing nearby) are connected with a single line. The red lines indicate the ETS gene clusters (genes reside tandem next to one another within a 200 kb genomic region).

To determine the distribution and duplication of the ETS genes in the genome, we searched the DNA sequence of each ETS genes in the genome database of each species and determined the chromosomal location of each ETS gene. Since the genome sequence of frog has not been assigned to individual chromosomes, we were not able to determine the chromosomal distribution of the ETS genes in frog. For all other species, the distributions of the ETS genes seem to be uneven among chromosomes, as having been observed for other gene families.^48^ Figure 3 illustrates the distribution of ETS genes in human genome (for other eight species, see Supplemental Fig. S4). Their chromosomal distribution patterns reveal that certain chromosomes and chromosomal regions have a relatively high density of ETS genes. For instance, in the human genome, four ETS genes are located on chromosome 1, whereas 12 chromosomes have no ETS gene at all.

There are some ETS genes that reside tandem next to one another. In this study, two or more ETS genes that occurred within a 200 kb genomic region were considered an ETS gene cluster. In Figure 3, these genes are marked with a red line. All species (except frog) considered in this study have one or more ETS gene clusters, and the larger the genome, the more such clusters. For example, the numbers of ETS gene clusters in human and nematode genome are five and one, respectively, which account for about 30% and 20% of the total ETS genes, respectively.

To detect the possible relationship between the ETS genes and the potential duplications of ETS genes in the genome, we constructed the phylogenetic tree for each species (data not shown). Genes at the terminal branches on the phylogenetic tree may represent recently duplicated genes.^43^ As shown in Figure 3, we identified 21 duplicated ETS genes (at nine terminal branches) in the human genome. Most of the duplicated genes are located on different chromosomes, a few of them are within a cluster defined duplicated ETS genes in other eight species are 17 in cattle, 20 in mouse, 20 in rat, 17 in chicken, 15 in zebrafish,15 in frog, 8 in fruit fly, 4 in nematode, and 4 in sea squirt (see Supplemental Fig. S4).

### Structure analyses of the human, cattle, and chicken ETS genes

**Figure 4:**
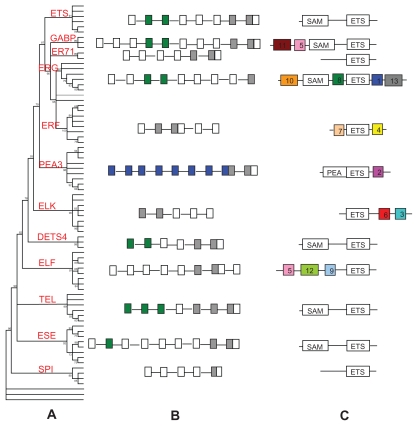
Clustering of 29 human, 27cattle and 20 chicken ETS genes by three methods. **A**) ML phylogenetic analysis reconstructed by phyML 3.0. **B**) Patters of exon-intron structure. Filled grey boxes: ETS domains; filled green boxes: SAM_PNT domains; filled blue boxes: ETS_PEA3_N domains; white boxes: other exon regions; lines; introns. **C**) MEME motif search results aligned based on the DNA-binding domains represented as white boxes. Conserved motifs are indicated in numbered color boxes. **Abbreviations:** SAM, SAM_PNT domain; PEA, ETS_PEA3_N domain; ETS, ETS domain.

We construct a ML phylogenetic tree (Fig. 4a) based on the ETS domain amino acid sequences of 29 human, 27 cattle, and 20 chicken ETS TFs. The topology of this tree is similar to that constructed using all 203 ETS TFs from the 10 species (Fig. 1). Furthermore, we analyzed the exon-intron structure of the human, cattle, and chicken ETS genes. Figure 4b shows the basic gene structural patterns of these group/subgroups. The genes in the same subgroup have similar structural pattern with minor exceptions in some subgroups. Exceptions occur in group SPI (gene hsets15), subgroups TEL (gene hsehts9), ETS (gene hssam8), and ERF (gene btets6). The results also reveal that all ETS genes have two or more exons which encode an ETS domain except those in group SPI and subgroup ERG. The details of the structures of all ETS genes of the three species are given in Supplemental Figure S1.

### Conserved motifs in ETS genes out off the conserved domains

We made MEME search of the conserved protein motifs flanking the ETS domain and other domains and uncovered 13 conserved motifs in the ETS TFs. As shown in Figure 4c, the ETS TFs in the same group (subgroup) share similar number and pattern of conserved motifs. The details of all conserved motifs of each ETS gene are given in Supplemental Figure S2. Each conserved motif appears only in one group (subgroup) except motif 3 and motif 5 that are found in two subgroups (ERG and ELK) and three subgroups (ELF, GABP, and ERF), respectively. The ETS TFs in group SPI, subgroups ESE, TEL, DETS4, ETS, and ER71 contain no such conserved motifs, but those in subgroup ELF contain eight motifs, three of which exist in the N-terminal of all ETS TFs in this subgroup, and the rest in the C-terminal of some proteins. For example, proteins hsets10, mmets6, rnets12, and btets4 contain motif 6 in their C-terminal, but the other ETS TFs do not.

### Function analysis of the ETS genes

The ETS TFs have been proved to be related to many biological processes. To understand the genome-wide functions of the ETS family, we used the online software DAVID to interpret its functions using gene ontology hierarchies. We uploaded the human, mouse, rat, chicken, fruit fly and sea squirt ETS gene list, and compared it with the existing reference gene list. The significant GO terms (FDR < 0.05) are shown in Table 3. The detailed p value and FDR value for each significant GO term in each species are given in Supplement Table S2. In the molecular function category, the significant GO terms include sequence specific DNA binding, nucleic acid binding, DNA binding, and transcription factor activity. In the biological process category, in addition to those which are in the common GO categories of transcriptional factors such as transcription, regulation of transcription and metabolic process, the ETS genes in the categories of RNA biosynthetic process, biopolymer metabolic process, macromolecule metabolic process also have highly significant enrichment annotation. Furthermore, positive regulation of transcription and cellular metabolic process also have highly significant enrichment annotation in the mouse ETS genes.

## Discussion

### Features of the ETS TFs

In this study, several features of the animal kingdom ETS TFs were revealed. First, the ETS TFs exist in all of the ten species studied. When searching for the ETS TFs in the yeast proteome, we did not find any homologues of ETS proteins. Several studies show that ETS TFs exist neither in plant, such as rice,^49^ Arabidopsis,^50^ and poplar,^51^ nor in bacteria and archaea.^52^ So, it seems that the ETS family is unique to metazoan animals, as suggested by Degnan^53^ and Laudet.^54^ Second, the number of ETS genes is proportional to the genome size. Although the numbers of ETS genes are different significantly in different lineages, the proportions of number of ETS genes to the total number of TF genes in lower organisms are similar to that in the higher organisms. The same phenomenon was also observed for non-ETS genes.^55^ Third, about 50% ETS TFs have either a SAM_PNT domain or a ETS_PEA3_N domain besides the ETS domain. The combination of DNA binding domains would be a source of generation of novel TFs, as suggested by Riechmann et al.^2^

### Duplications of the ETS genes

Duplication at both gene and genome levels is a pervasive process and contributes to the origin of biological novelty in evolution.^56^ Duplications on genome level are thought to have occurred throughout the process of animal and plant evolution.^57^–^59^ Xiong^43^ analyzed TFs of the rice genome, and found twelve pairs of large duplicated segments which account for ~45% of the rice genome. About 62% (991) of the 1611 TF genes identified in rice reside in the duplicated segments, of which 592 are retained as duplicated pairs. From the phylogenetic tree for each of the ten animal species, we found that the duplicated ETS genes account for 69% of the total ETS genes, ranging from 36.4% in Sea squirt to 85% in chicken. High proportions of duplicated genes were also reported in other TF families, e.g. ~60% in GATA family in Arabidopsis.^18^

In addition, we also observed an interesting phenomenon. In all studied endotherm animals (human, cattle, mouse, rat, and chicken), an ETS gene cluster located on one chromosome is duplicated on another chromosome. For example, in human a cluster (containing genes hssam2 and hssam10) residing on chromosome 11 at position q23.24 is duplicated on chromosome 21 at position q22 (containing genes hssam8 and hssam1). This kind of duplication can be used as a support for the vertebrate specific block duplication event, leading to increase of various paralogous copies of genes.^60^,^61^

### Functional divergence of ETS genes

The difference in gene structure and amino acid sequence among different subgroups provide us with some hints that ETS genes may have a variety of physiological functions. The variety of subgroups within group II reflects a big spectrum of structural and functional diversity of this group. It has been found through cell culture that some ETS proteins, e.g. those which are classified into group SPI and subgroups ELF, ETS, and ERG preferentially expressed in cells which are developed from mesoderm, such as hematopoietie, vascular endothelial, kidney, intestine, and liver cells.^62^ Sharrocks^4^ proved that the ETS TFs were involved in various processes during embryonic development in several organisms, such as fruit flies, worms, fishes, frogs and mice. Multiple ETS factors have been found to be associated with cancer. For example, the ERG ETS TF is fused to the EWS gene.^4^ Many ETS TFs are known to represent nuclear targets of signaling pathways.^63^ Some ETS-domain subfamily play key role in immune system.^4^ Gene function prediction of the mouse and human ETS TF family was performed in this study. Besides the common GO categories of TFs, many ETS TFs in mouse have significant enrichment annotation in categories of cell cycle, organ development, and cell differentiation. However, the categories of immune system process and immune response do not have significant enrichment annotation.

The overall conservation of protein sequences often implies the similar molecular and biological functions of them. The ETS TFs of the same group or subgroup usually have equivalent or similar biological functions. For example, the members in group SPI, including three human, three mouse, three rat, two cattle, two chicken, two zebrafish, three frog, and one nemolade ETS genes, were reported to be involved in immune system such as B-cell function and myeloid and lymphoid differentiation.^4^ However, some ETS TFs of the same group play different biological roles. For example, genes drpea1, hspea1, and mmpea4 (named pea3 in NCBI) and genes ggpea1, hspea2, and mmpea3 (named er81 in NCBI) in subgroup PEA3 are involved in muscle differentiation and directing sensory-motor neuron connections, respectively; genes mmsam7, rnsam10, and hssam10 (named ETS1 in NCBI) and genes ggsam3, hssam8, mmsam4 (named ETS2 in NCBI) in subgroup ETS are related to T-cell survival and hair development, respectively; genes hsets5 and mmets10 (named Sap1 in NCBI) and gene ceets5 named lin1 in NCBI in subgroup ELK participate in T-cell differentiation and vulval development, respectively.^4^ Moreover, some particular ETS genes possess multiple biological functions, for example, genes ggsam3, hssam8, mmsam4 (named ETS2 in NCBI) plays roles in extraembryonic tissue generation, extracellular matrix remodeling, and hair development; genes ggsam5, hssam7, mmsam10 (named Tel in NCBI) plays roles in yolk-sac angiogenesis and adult haematopoiesis.^4^

### Evolution of the ETS genes

We constructed the molecular phylogenetic tree of the ETS TF family for ten species of animal kingdom. The overall divergence pattern of the ETS genes appears similar to that of other gene families such as the homeobox family^12^ and the nuclear receptor genes.^13^ Laudet et al^30^ constructed a phylogenetic tree of the ETS gene family using 61 known ETS genes and showed the ETS TF family members can be classified into 13 groups, which could be further clustered into five subfamilies. Our classification is in general consistent with theirs. The ETS genes which were included in both studies were all classified into same groups except that the gene *Drosophila* YAN (named dmsam5 in our study) was classified into group YAN which contains only this gene, while in our study it was classified into group TEL. In addition, the three nematode genes, ceets1, ceets4 and ceets8, which were not included in Laudet et al,^30^ were classified into one subgroup named CEETS. So, the total number of subgroups in group II is the same in both studied. Furthermore, in our phylogenetic tree, the 12 subgroups in group II are all in relatively independent branches, while in their phylogenetic tree, groups ETS, ER71, GABP, PEA3, ERG, ERF and ELK are merged into a large branch and thus are classified into one subfamily (named ETS), similarly, groups ELF and ESE are classified into another subfamily (named ELF), and the other groups are in different individual subfamilies.

The ETS TFs within each subgroup generally contain the same domain combination. With only minor exceptions, the ETS TFs in subgroups ELF, ER71, ELK, CEETS and ERF contain only one ETS domain, those in subgroup PEA3 contain both ETS and ETS_ PEA3_N domains, and those in subgroups ESE, TEL, DETS4, and ETS contain both ETS and SAM_PNT domains). So, we infer that the ancestor gene of group II might have duplicated into three copies. The first copy might evolve to subgroups ELF, ER71, ELK, CEETS and ERF, the second to subgroup PEA3, and the third to subgroups ESE, TEL, DETS4, and ETS. A confused situation is that in subgroup ERG about 2/3 ETS TFs contain both ETS and SAM_PNT domains and the rest contain only one ETS domain.

Our results show the ETS genes of mammalian animals exist in both groups I and all subgroups of group II. So, we infer that the diversification of these genes predates the divergence of mammalian animals. Moreover, Degnan^53^ and Laudet^30^ suggested that the diversification of the ETS TF family was already achieved before the separation of the major phylum of metazoans. We deem that the question of the origin of the ETS genes remains open, and that it would be interesting to investigate the ETS genes in other lower metazoan animals.

## Figures and Tables

**Table 1:** ETS TF subfamilies and distribution of ETS TFs in different subfamilies and in the ten species of animal kingdom. First row: numbers of proteins, second row: numbers of genes encoding these proteins.

	Subfamily*									
Species	ETS	ETS& SAM_PNT	ETS& ETS_PEA3_N	ETS + 2	ETS& DNA_pol_B	ETS& ETS_PEA3_N + 2	ETS& AT_hook& SAM_PNT	Total No ETS TFs	Total No TFs	ETS TFs/TFs (%)
Human	30	34	7	0	0	0	0	71	2740	2.6
	15	11	3					29	1453	2.0
Mouse	24	20	7	0	0	0	0	51	2416	2.1
	14	10	4					28	1377	2.1
Rat	19	13	4	0	0	0	0	36	1669	2.1
	15	10	3					28	1167	2.4
Cattle	15	10	3	0	1	0	0	29	1381	2.1
	14	10	3		1			28	1136	2.5
Chicken	9	15	5	0	0	1	1	31	1084	2.9
	7	10	3			1	1	22	720	3.1
Zebrafish	19	11	4	0	0	0	0	34	1853	1.8
	11	7	3					21	1234	1.9
Frog	11	8	1	0	0	0	0	20	981	2.0
	11	8	1					20	981	2.0
Sea squirt	8	12	0	0	0	0	0	20	518	3.9
	4	7						11	374	3.7
Fruit fly	11	7	0	0	0	0	0	18	963	1.9
	6	5						11	579	1.9
Nematode	9	1	0	1	0	0	0	11	1159	0.9
	8	1		1				10	793	1.3
Total	155	131	31	1	1	1	1	321		
	104	79	20	1	1	1	1	207		

* ETS TFs are classified into different subfamilies according to the DNA-binding domains they contain. For example, ETS TFs in subfamily ETS contain only an ETS domain, those in subfamily ETS&SAM_PNT contain an ETS domain and a SAM_PNT domain, those in subfamily ETS + 2 contain two ETS domains, et al.

**Table 2:** Core conserved sequence of the ETS domain in different (sub)groups in the ML tree.

Group	Sequence logo	Group	Sequence logo	Group	Sequence logo
SPI	MTYQKMARALRNYG	ESE	MTYEKLSRALRYYY	TEL	MTYEKMSRALRHYY
ELF	MNYETMGRALRYYY	DETS4	MNYDKLSRSLRQYY	PEA3	MNYDKLSRSLRYYY
ELK	MNYDKLSRALRYYY	ETS	MNYEKLSRGLRYYY	GABP	MNYEKLSRALRYYY
ERF	MNYDKLSRALRYYY	ERG	MNYDKLSRALRYYY	CEETS	MNYDKMSRGLRYFY

**Table 3:** The significant gene ontology (GO) terms of the ETS gene (FDR < 0.05).

Category	GO term	GO definition	Species
Molecular Function	0043565	sequence-specific DNA binding	human, mouse, rat, chicken, sea squirt, fruit fly
	0003676	nucleic acid binding	human, mouse, rat, chicken, fruit fly
	0003677	DNA binding	human, mouse, rat, chicken, fruit fly
	0003700	transcription factor activity	human, mouse, rat, chicken, fruit fly
	0030528	transcription regulator activity	human, mouse, rat, chicken, fruit fly
	0016563	transcription activator activity	human, mouse
Cellular Component	0005634	nucleus	human, mouse, rat, chicken
	0043231	intracellular membrane-bound organelle	human, mouse, chicken
	0043227	membrane-bound organelle	human, mouse, chicken
	0043229	intracellular organelle	human, mouse
	0043226	organelle	human, mouse
	0044424	intracellular part	human, mouse
	0005622	Intracellular	mouse
Biological Process	0006350	Transcription	human, mouse, rat, chicken, fruit fly
	0010467	gene expression	human, mouse, rat, chicken, fruit fly
	0010468	regulation of gene expression	human, mouse, rat, chicken, fruit fly
	0019219	regulation of nucleobase, nucleoside, nucleotide and nucleic acid metabolic process	human, mouse, rat, chicken, fruit fly
	0019222	regulation of metabolic process	human, mouse, rat, chicken, fruit fly
	0050789	regulation of biological process	human, mouse, rat, chicken, fruit fly
	0050794	regulation of cellular process	human, mouse, rat, chicken, fruit fly
	0031323	regulation of cellular metabolic process	human, mouse, rat, chicken, fruit fly
	0006139	nucleobase, nucleoside, nucleotide and nucleic acid metabolic process	human, mouse, rat, chicken
	0006351	Transcription, DNA-dependent	human, mouse, rat, chicken
	0006355	regulation of transcription, DNA-dependent	human, mouse, rat, chicken
	0016070	RNA metabolic process	human, mouse, rat, chicken
	0032774	RNA biosynthetic process	human, mouse, rat, chicken
	0045449	regulation of transcription	human, mouse, rat, fruit fly
	0065007	biological regulation	human, mouse, chicken
	0043283	biopolymer metabolic process	human, mouse, chicken
	0044237	cellular metabolic process	human, mouse
	0044238	primary metabolic process	human, mouse
	0043170	macromolecule metabolic process	human, mouse
	0006357	regulation of transcription from RNA polymerase II promoter	human, mouse
	0008152	metabolic process	human, mouse
	0006366	transcription from RNA polymerase II promoter	human
	0045935	positive regulation of nucleobase, nucleoside, nucleotide and nucleic acid metabolic process	mouse
	0045941	positive regulation of transcription	mouse
	0031325	positive regulation of cellular metabolic process	mouse
	0009893	positive regulation of metabolic process	mouse
